# Stress Tolerance of Bed Bugs: A Review of Factors That Cause Trauma to *Cimex lectularius* and *C. Hemipterus*

**DOI:** 10.3390/insects2020151

**Published:** 2011-04-29

**Authors:** Joshua B. Benoit

**Affiliations:** Division of Epidemiology of Microbial Diseases, School of Public Health, Yale University, New Haven, CT 06510, USA; E-Mail: joshua.benoit@yale.edu; Tel.: +1-203-737-4134.

**Keywords:** stress tolerance, traumatic insemination, bed bug, *Cimex*, cold, heat, dehydration

## Abstract

Recent emergence of bed bugs (*Cimex* spp.) has prompted a significant expansion of research devoted to this pest. The ability to survive and recover from stress has significant implications on the distribution and survival of insects, and bed bugs are no exception. Research on bed bug stress tolerance has shown considerable progress and necessitates a review on this topic. Bed bugs have an extraordinary ability to resist dehydration between bloodmeals, and this represents a critical factor allowing their prolonged survival when no host is available. High relative humidities are detrimental to bed bugs, leading to reduced survival in comparison to those held at lower relative humidities. Continual exposure of bed bugs, eggs and mobile stages, to temperatures below freezing and short term exposure (=1 h) to temperatures below −16 to −18 °C results in mortality. The upper thermal limit for short term exposure of eggs, nymphs and adults is between 40–45 °C for the common (*Cimex lectularius*) and tropical (*C. hemipterus*) bed bugs. Long-term exposure to temperatures above 35 °C results in significant reduction in survival of mobile bed bugs. Eggs for *C. lectularius* and *C. hemipterus* are no longer viable when held below 10 °C or above 37 °C throughout embryogenesis. Blood feeding, although necessary for survival and reproduction, is discussed as a stress due to thermal and osmotic fluctuations that result from ingesting a warm bloodmeal from a vertebrate host. Cold, heat, water stress and blood feeding prompted the expression of heat shock proteins (Hsps). Pesticide application is a common human-induced stress for urban pests, and recent studies have documented pesticide resistance in many bed bug populations. High levels of traumatic insemination (mating) of bed bugs has been linked to reduced survival and fecundity along with possibly exposing individuals to microbial infections after cuticular penetration by the paramere (=male reproductive organ), thus represents a form of sexual stress. Additionally, less common stress types such as microbial infections that have been documented in bed bugs will be discussed. Overall, this review provides a current update of research related to bed bug stress tolerance and how their ability to resist stressful conditions has lead to their expansion and proliferation.

## 1. Introduction

Bed bugs have experienced a worldwide emergence in the last decade, particularly in regions that were once rid of this pest [1,2]. Humans have been plagued by bed bugs since at least 1350 BC as evidenced by their recovery from Egyptian archeological sites [3,4]. Bed bugs are Old World pests that were introduced to the Americas by early immigrants and were endemic before World War II [2]. Wide spread application of dichloro-dephenyl-trichloroenane (DTT) and other residual pesticides resulted in the near eradication of bed bugs throughout North America [2,5]. Over the past decade, bed bug populations have increased worldwide and are now present in nearly all major cities [6–10]. The exact cause of this emergence is not known, but is likely due to a combination of increased travel, frequent exchange of used, potentially bed bug-infested furniture, increased usage of species-specific bait traps over residual pesticide spraying and the increased pesticide resistance in bed bug populations [11–17].

Previous studies have failed to conclusively prove that bed bugs can act as a disease vector [2,18–20]. The negative consequences of bed bugs are more due to reduction in quality of life from a combination of health and economic impacts. Individuals that are allergic can experience severe irritation along with erythematous or papular urticaria-like dermatitis, and these can lead to secondary infections [21–26]. Along with the direct effects of bed bug bites, infestations can cause anxiety and insomnia [20,26,27]. Bed bug infestations have been documented in different socio-economic classes, but large infestations are more prevalent in low income housing. This is likely due to the lack of resources, *i.e.* insufficient funds for pest control, to respond to infestations. The substantial monetary resources necessary to eliminate bed bug infestations suggest that economic consequences may be more critical than health-related issues [21–27].

Studies on biochemistry, physiology and molecular biology have been minimal or lacking since 1960, which is likely due to the eradication of bed bugs from most developed countries [1,28]. The recent bed bug emergence has prompted research in all areas of bed bug biology. In particular, there have been multiple studies on the molecular physiology of the common bed bug, *Cimex lectularius*, including the regulation of heat shock proteins [29], genetic basis of pesticide resistance [16,28,30] and the bed bug sialome [31]. Additionally, recent studies have begun to examine many physiological aspects of the common and tropical (*C. hemipterus*) bed bugs including more in-depth understanding of positive and negative effects of traumatic insemination [32–38], effects of blood feeding as a stress and comparative aspects of environmental stress tolerance [29,39,40].

There have been many studies that focused on aspects of bed bug stress tolerance, particularly abiotic stresses such as cold, heat and dehydration, but no reviews have provided a complete synopsis of the response of bed bugs to stress. This article presents a review of research on bed bug stress tolerance emphasizing recent advances that focus on the common bed bug, *C. lectularius*, and the tropical bed bug, *C. hemipterus*. First, a section on the basic biology of bed bugs is provided, including their development and reproduction, host seeking and blood feeding, bed bug chemical ecology and population aggregation and dispersal. More detailed information on general bed bug biology is provided by Reinhardt and Siva-Jothy [1] and Usinger [2]. Second, background on general insect stress tolerance is discussed, specifically behavioral, biochemical and physiological mechanisms that allow individuals to avoid, tolerate and recover from exposure to adverse conditions. Lastly, there is a synopsis of current research on bed bugs during exposure to environmental stress, blood feeding, traumatic insemination, pesticide application and other less common stresses.

## 2. Bed Bug Biology

### 2.1. Development and Reproduction

The development of bed bugs is similar to that of other hemimetabolous insects, specifically other hemipterans [2]. Briefly, bed bugs progress through five nymphal instars before emerging as adult males and females [2]. Usually each stage requires a bloodmeal to progress to the next instar, and development is dependent on temperature and host availability [2,41]. Under warm, favorable conditions (25–32 °C, 40–75% RH) bed bugs can complete development in a little over one month and under cool conditions (below 20 °C) development can take one-two years [2,29,41–43]. As with development, longevity is dependent on temperature and access to blood, where fifth instar nymphs and adults can persist up to two years under the proper conditions with no access to blood [2,29,41–43].

The reproductive physiology of bed bugs has been well studied [1,2,33,38]. First, bed bugs need to locate the opposite sex. Likely, individuals are drawn to bed bug harborages by the presence of aggregation pheromones [44]. Once aggregated, males seem to be attracted and copulate with any object the size of bed bugs [2,33,41,45]. After mounting the dorsal side of the female, the male probes the female with his intermittent organ (= paramere) until locating the penetration site (female ectospermalage) on the ventral side of the abdomen to deposit sperm [46]. This process of mating is known as traumatic insemination, and represents a period of stress due to sexual conflict [33]. The role of traumatic insemination as a stress is discussed later in this review. Females become sterile after 35–50 d of isolation, indicating that multiple matings are necessary throughout the lifetime of the female [2,32,46–48]. Egg laying will occur until senescence (30–250 d depending on species, 200–250 d for *C. lectularius*; [2,32,38]) as long as females can periodically mate and blood feed. Egg production, rate and total eggs laid, is dependent on temperature and access to blood [2,41]. Johnson [41] provides an excellent review of the interplay between temperature, blood feeding and egg production for *C. lectularius*.

### 2.2. Host Location and Blood Feeding

All cimicids are obligate hematophages, obtaining nutrients from only the blood of their host. Thus, all stages and both sexes require vertebrate blood to survive, develop and reproduce [2]. The evolution of blood feeding in cimicids likely occurred only once, but how it exactly evolved has not been determined [49]. Feeding usually occurs weekly, if a host is available, and the bloodmeal represents a 1.5–6.1× increase in unfed body mass [2]. Cimcids usually have a narrow range of hosts that share some particular ecological features such as predictable distribution, both temporal and spatial, assembly in enclosed spaces (cave, buildings, *etc.*) and warm body temperature [1]. Likely, cimcids initially parasitized bats or birds, and then made the transition to bats, birds and humans due to coexistence [2]. Currently, there have been three species of cimicids that have made the switch to using humans as the primary host, *C. lectularius, C. hemipterus,* and *Leptocimex boueti* [2]. The trigger for changing to a new host is likely starvation from the absence of the primary host. If fitness while feeding on a new host is comparable or better to that when utilizing an old host, selection will allow the individuals to remain utilizing the new host. The major factor that determines if transfer will occur is if the stylet morphology (specifically the food canal), feeding ability and digestive system developed for the previous host is compatible with the new host. As an example, the diameter of the *C. lectularius* food canal is 8–12 μm [50], which can accommodate feeding on chicken erythrocytes (∼11.2 μm in diameter) and human erythrocytes (6–8 μm in diameter). In *C. hemipterus*, flexibility of the joint and hinge system of the mouthparts allows *C. hemipterus* to control food canal size for access to different hosts and similar mechanisms likely act for *C. lectularius* [2]. The flexibility of the stylet and differences in erythrocyte sizes may be responsible for the drastic differences in the feeding time and egg production when bed bugs utilize different hosts [2].

As mentioned before, blood is the only source of nutrients for bed bugs. Although rich in protein and other resources, blood is devoid of key nutrients, such as B vitamins [49]. This is remedied by the harboring symbiotic bacteria within the mycetome (a paired organ located adjacent to the gonad [2,51]). Recent studies on *C. lectularius* have identified that *Wolbachia* and γ-proteobacteria are the bacteria present in the mycetome [52]. Elimination of *Wolbachia* from the mycetome by antibiotic treatment resulted in reduced fecundity that can be rescued by vitamin supplements [52]. The similarity between *Wolbachia* in *C. lectularius* and other cimicids suggests the function of this bacterium is likely conserved among Cimicidae [53,54]. Additionally, there are at least two other symbionts that are present within *C. lectularius* [51,55–57], but the exact role of these bacteria on bed bug physiology is unknown. Heat exposure reduced fecundity of *C. lectularius* by 90%, likely due to changes in microbes within the mycetome [55]. Reduction in the mycetomes may be involved in bed bug senescence since this organ is not present or greatly reduced when females cease laying eggs [41,56].

Host location is extremely important to the survival of bed bugs since egg production depends the acquisition of a bloodmeal and first instar nymphs need to feed within a few days after emergence since starvation and dehydration occur quickly [2,29,39,41–43]. Locating of a host occurs in a three phases: (1) searching, (2) orientation to host, and (3) contact with host [49]. First, Romero *et al.* [58] have studied the spontaneous locomotor activity of bed bugs. In this study, activity normally increased during the scotophase with starved adults moving more frequently than recently fed individuals [58]. As starvation proceeds, bed bugs transition to a host cue dependent search strategy to conserve nutrients [58]. Host cues that have been identified that operate at a longer distance (∼1.5 m) include temperature, CO_2_ and other host kairomones [45,59–62]. Once the host has been located, temperature of and kairomones on the skin elicit probing with the proboscis [2,59–61,63]. After engorgement, bed bugs become repelled or are no longer attracted to host cues [59,60].

### 2.3. Bed Bug Pheromones

Multiple pheromones have been identified from bed bugs, including different types of alarm pheromones and the airborne aggregation pheromone [44,61,64–70]. The alarm pheromone of bed bugs consist of (E)-2-hexenal, (E)-2-octenal, 4-oxo-(E)-2-hexenal and 4-oxo-(E)-2-octenal [65,68,70]. (E)-2-hexenal and (E)-2-octenal were the first to be isolated and prompt the typical alarm response [40,70]. 4-oxo-(E)-2-hexenal and 4-oxo-(E)-2-octenal have only recently been recovered from dorsal abdominal glands of 5th instar nymphs and act as alarm pheromones [68,70]. The activity of the aggregation pheromone was identified by Levinson and Bar Ilan [64], but only recently was the chemical composition identified by Siljander *et al.* [44]. Antennal sensing of pheromone and other chemical cues has been thoroughly characterized in *C. lectularius* [69]. A recent summary by Weeks *et al.* [61] provides a comprehensive review of bed bug chemical ecology.

### 2.4. Population Dispersal, Localization and Aggregation

Bed bugs usually reside within protective harborages near their host [2]. These sites are maintained by the presence of chemical cues, likely aggregation pheromones [1,2,44,71]. Infestation rates of different regions vary greatly, and for humans, localities with high turnover rates and brief occupancy are more prone to bed bug outbreaks [1,2,5,72,73]. Thus, people that are highly mobile such as backpackers, immigrants, and homeless people, are frequently associated with passive transport of bed bug in their clothing, luggage and furniture [2,5,6,74,75]. In relation to agriculture, bed bugs have been commonly associated with poultry breeding facilities [76]. Active dispersal is accomplished by individual bed bugs walking between rooms, usually after extended periods without a host available at close range, following the release of alarm pheromones after colony disturbance that prompt harborage evacuation or after exposure to certain pesticides [2,41,77]. Overall, long distance mobilization of bed bugs between buildings, cities and countries is likely accomplished by passive transport and active movement of bed bugs is responsible for movement within or between adjacent buildings.

## 3. Insect Stress Tolerance

Insect stress tolerance has been the subject of many reviews including heat [78,79], cold [80–83], water stress [84–87], changes in the expression of stress proteins [88,89], microbial infections [90–94] and diapause [95,96], thus there is a great deal of literature on this topic. These previous general reviews could provide background for research directed towards bed bug stress tolerance. Overall, any factor that results in potentially negative consequences to an insect can be classified as stress. This includes both abiotic factors such as temperature and water stresses and biotic factors such as fungal and bacterial infections. Briefly, stress is signaled by an indicator, such as membrane damage, misfolded proteins or DNA damage, which begins the stress response. Cellular damage that is too severe for recovery will usually result in necrosis (uncontrolled cell death) or apoptosis (controlled cell death). Following cellular survival, the stress signal progresses by stress-activated protein kinase pathways (SAPK). Detailed reviews of these pathways in relation to insect stress tolerance have been previously organized by Stronach and Perrimon [97] and Hatanaka et al [94]. SAPK pathways are typical mitogen activated protein kinase (MAPK) pathways, and Jun Kinase (JNK) and p38 pathways are the two most common [94,97–100]. These two pathways have been linked to multiple types of stress in many insects [101–107], and are likely associated with stress signaling in bed bugs.

Many proteins have been associated with insect stress either by their upregulation or through gene knockdown experiments [89,108–110]. The most studied class of proteins involved during insect stress is heat shock proteins (HSPs), which are predominantly classified as chaperones [88,89]. These stress proteins have been examined in bed bugs in relation to environmental stress [29] and blood feeding [111] and are discussed later in sections on environmental stress tolerance and blood feeding. Antioxidants have been documented in response to many types of insect stress to prevent oxidative stress [89]. Ice active proteins (IAPs) have been shown to be critical for cold and freezing tolerance [112]. Along with heavily-studied stress proteins, many others have been documented to be critical to stress tolerance, but their primary role is not during the stress response [89]. As an example, expression of aquaporins, a water channel protein, is critical to during cold exposure, dehydration tolerance and removal of excess fluid during feeding [113–115]. Cytoskeletal proteins have been noted to be important during cold exposure, dehydration stress and blood feeding [87,89,116]. Lastly, there have been many proteome and transcriptome studies focused on the response of insects to stress, particularly cold, heat and dehydration [89,108–110]. These have led to the identification of many genes that are potentially critical for tolerating and responding to stress.

Changes in particular metabolites have been observed during stress exposure [89]. It is important to note that insects can tolerate large concentration ranges of sugars and other biochemical molecules in their hemolymph [117–119]. The ability of insects and other invertebrates to use increases in these metabolites to reduce damage from stress is unique, as other organisms, particularly mammals and other vertebrates, cannot tolerate high concentrations of particular metabolites, such as sugar. Currently, two molecules, trehalose and glycerol, have been studied in multiple insect systems [117,120–123]. Trehalose increases have been documented during cold and dehydration exposure [118,121–123] and likely act as a buffer to prevent unwanted biochemical interactions [117,123–126]. Glycerol has been documented as a cryoprotectant and desiccation buffer [117]. Many other metabolites have been identified to increase during stress exposure [121,122], and additional studies will be needed to determine the exact role of each during stress.

Studies have recently highlighted significant metabolic changes after stress. Usually, there is reduction in metabolic proteins during or immediately following the stress and a subsequent increase in these proteins during the recovery period [83,108–110,127–129]. For thermal resistance, studies indicate that metabolic genes and proteins are expressed at lower levels immediately after stress and increase during recovery [107–110,127,130–132]. Decreases in metabolism have been documented during dehydration, particularly as a method to suppress water loss through respiration and reduce oxidative stress [87,129,133–136]. Overall, metabolism suppression during stress prevents damage from the generation of excess reactive oxygen species (ROS) associated with metabolism during a period when individuals are responding to damage caused from stress exposure.

Rather than only responding to stress, insects can prevent stress by avoidance, thus reducing the energetic demands of repairing stress-induced damage. Long-distance migration into favorable regions occurs in few insects, such as Monarch butterflies. Another possibility at avoidance is to enter a period of dormancy [95,96] and retreat into protective harborages [79,95,133]. Within these refuges, environmental changes are buffered to reduce temperature fluctuations and the localized relative humidity is much higher [133]. Clustering is utilized to prevent water loss within harborages, and this has been noted in bed bugs [39]. As the group size increase, water loss rates drop in the individual leading to enhanced water conservation [39,117,137]. Thus, behavioral changes represent a mechanism by which insects can prevent stress.

Recently, multiple bouts of stress exposure have been identified to be rife with negative consequence [138,139]. For many years, studies focused on one bout of stress, rather than multiple exposures [138–141]. Numerous studies have shown that multiple freeze/thawing or dehydration/rehydration bouts have negative impacts that compound with each cycle [138–141]. During each exposure, individuals need to utilize a finite amount of nutrients, leading to the inability to respond as the number of bouts increase. These results suggest that multiple bouts of stress need to be assessed when examining insect stress tolerance.

## 4. Bed Bug Stress Tolerance

Currently, studies on bed bug stress tolerance have been lacking with the exception of early projects before 1960 when bed bugs were more prevalent [1,2]. Only recently studies have begun to identify aspects that cause stress along with mechanisms that promote tolerance and potential response factors to stress in bed bugs.

### 4.1. Environmental Stress Tolerance

Studies on water balance of bed bugs have revealed that common and tropical bed bugs are extremely resistant to dehydration [29,39,42] (Table 1). The water loss rates for all stages of *C. lectularius* were extremely slow [39] and comparable to other insects that are extremely resistant to dehydration [85–87]. First instar larvae are the developmental stage that is least resistant to dehydration and die quickly under dry conditions with no bloodmeal [29,39,42]. Fifth instar nymphs and adults are the most resistant to dehydration, which is likely only due to surface to volume ratios which decrease as bed bugs advance instars [29,39,42]. Along with differences in dehydration resistance between stages, high temperature increases water loss substantially, leading to a significant reduction in the length of survival under dehydrating conditions [29,39,42]. Exposure to alarm pheromones was shown to reduce the ability of bed bugs to maintain water balance [40]. Bed bugs are vulnerable to overhydration with reduced survival following prolonged exposure to conditions near saturation (100% RH) [29,39,42,142]. Immersion of mobile stages of *C. lectularius* in liquid water for 24h results in significant mortality, but this treatment had no discernable consequence on eggs [143]. Dehydration has been documented to increase transcript levels for heat shock protein 70 (Hsp70) and Hsp90 after 5d under 0% RH and during rehydration at 100% RH [29].

The upper thermal limits for tropical and common bed bugs have been determined (Table 1). Generally, *C. hemipterus* is slightly more resistant to heat than *C. lectularius* [29,39,42]. This is not surprising since tropical species are usually more tolerant to higher temperatures than related temperate species. For eggs, incubation length decreases with increasing temperature [2,41,42]. The incubation period of *C. hemipterus* eggs decreases until up to 35–37 °C, but over 37 °C hatchability is reduced until no eggs are viable at 37 °C [42]. No *C. lectularius* eggs are viable if held above 37 °C [2,14]. At the lower limit, bed bug eggs are no longer viable if individuals are held below 10 °C throughout embryogenesis, and short term exposure to temperatures near −15 °C will reduce egg viability [2,42,143].

Mobile stages of bed bugs have a relatively high heat tolerance (Table 1). For the common and tropical bed bug, short term heat tolerance (∼1 h exposure) can reach 46 to 48 °C, but continual exposure around 40 °C results in significant reduction of longevity and survival [2,29,42]. In comparison to the heat exposure research, cold temperature exposure has not been as thoroughly studied [2,29]. Bed bugs are not tolerant of freezing and adult females have a super cooling point (SCP, the temperature that insect freezing occurs) at −20 °C to −21 °C [29]. Adult females of *C. lectularius* can tolerate −14 °C to −16 °C for short periods with increased cold tolerance after rapid cold hardening, a short term pre-exposure to warmer than lethal temperatures that promotes survival when individuals are moved potentially lethal temperatures [29]. For all stages of *C. hemipterus* and *C. lectularius*, prolonged exposure to temperatures below freezing has been shown to be lethal [2], but in depth studies will be needed to assess cold tolerance for each developmental stage. Temperatures below 10 °C prevent molting to the next developmental stage [2,42]. Expression of Hsps increased in response to cold and heat in *C. lectularius* [29], indicating a role for these proteins in bed bug temperature tolerance.

The utilization of cold and heat exposure has been touted to be a potential method for the control of bed bugs [143,146]. Based on heat tolerance studies, short term exposure (∼1 h) to temperatures above 46 °C or extended time at slightly lower temperature will be useful for eradication of bed bug populations [29,42]. Laundering of bed bug infested materials in warm water or extended periods in a dryer can kill bed bugs [143]. To utilize cold for control, bed bugs may need to be exposed to temperatures slightly below freezing for much longer periods (3–5 d) to ensure bed bug death. Thus, time and temperature of exposure need to be carefully examined before using cold and heat exposure for bed bug control, especially when bed bugs may be protected from thermal changes within their off-host harborages.

### 4.2. Blood Feeding

Blood feeding, although necessary for survival and reproduction, can be an extremely taxing period [49,89,111]. In particular, blood feeding insects needs to evade the host's immune response [49], tolerate significant changes in water content due to fluids in the bloodmeal [49,89], and respond to the temperature changes due to the heat of the bloodmeal [111]. Hematophagous insects have adapted to tolerate these conditions to utilize the protein- and lipid-rich blood [49]. To resist the rapid overhydration, nearly all blood feeding insects, including bed bugs, have developed efficient excretory systems to remove the excess fluid in the bloodmeal [147–150]. Temperature changes following blood feeding in insects can potentially be 10–12 °C, prompting the heat shock response to protect the midgut from heat stress [111]. This response has been documented in bed bugs [111]. Overall, although blood feeding is necessary for bed bug development, survival and reproduction, it is still a drastic physiological shift that likely results in multiple stress types.

### 4.3. Traumatic Insemination

Bed bugs are polyandrous insects (female mate with multiple males) and optimal mating interaction for the male is not same as the female [1,2,33]. This is due to bed bugs undergoing traumatic insemination and male sperm precedence [2,46]. Briefly, male reproductive organ (=paramere) is heavily sclerotized to allow the male to copulate by piercing the abdominal wall of female and transferring sperm directly into the hemocoel, even though the female genital tract is intact [1,2,33]. To tolerate insemination from the male, females have developed supplemental mesodermal genitalia (=paragenitalia [33]). There is a wide-range of adaptations among cimcids, ranging from almost non-existent paragenitalia (*Primicimex cavernis*) to others with extensive paragenitalia (*Crassicimex sexualis* [33]). In *C. lectularius* and *C. hemipterus*, the most distinct aspect of the paragenitalia is the spermalage [1,2]. This organ is noticeable on the surface of the female as an ectospermlage, a dorso- ventral groove in the fifth abdominal sternite. This groove likely serves as a guide for males during mating so the paramere pierces the female abdomen in favorable areas. The mesopermalage, a bag structure full of hemocytes (blood cells associated with immunity and wound healing), is directly under the ectospermalage. Males inject semen directly into the mesospermalage during copulation and the sperm migrates through the hemolymph until penetration at the oviduct allowing fertilization after movement to the ovaries [2]. It is not uncommon that females mate multiple times with different males, even though this may be unfavorable [32,33]. This is due to precedence for sperm of the last male to copulate to fertilize more eggs [2,32]. Thus, even though mating is necessary, the act of traumatic insemination is fraught with potential aspects that can cause stress.

**Figure 1:**
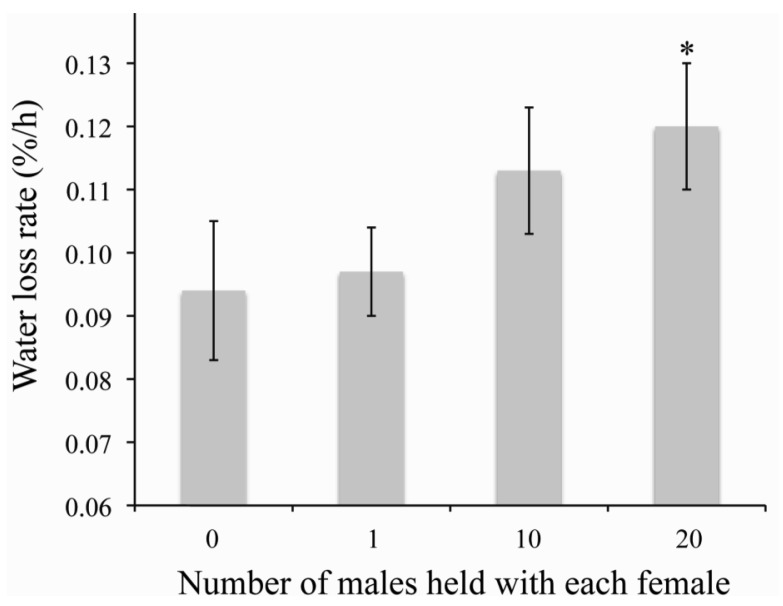
Proportion of water mass lost at 0% relative humidity (RH) and 25 °C after removal of an individual female held in containers containing 0, 1, 10 and 20 males for 6 h. Water loss rates were determined according to Benoit *et al.* [39]. Water loss rate is presented as percent water lost per hour. Data represents the mean ± SE, N = 30.*, significantly different from 0 males (P < 0.05).

There are three main negative consequences of traumatic insemination, (1) reduced longevity and reproductive success of the females, (2) potential infections due to piercing of the paramere into the female and (3) unwanted copulation between fifth instar nymphs/males and other conspecific males [32,33,38]. Experimental manipulation of mating indicates that high mating frequencies, considered normal, reduces the longevity of bed bugs by nearly 40d [32]. The longer survival of female bed bugs due to low mating increased egg production throughout the lifetime of a female [32]. Although low mating is preferable to females, sperm precedence indicates that multiple matings are beneficial to males to ensure that it is the last male to mate [32,33]. The exact mechanism for this reduced longevity is not known. Two possibilities for the reduced lifespan after mating are nutrient resources need to be utilized for repairing the damage caused by copulation and insemination or cuticle piercing increases the likelihood of microbial infection [33,34,151]. The spermalage is likely present to reduce the likelihood of microbial infections during traumatic insemination [34]. Along with these possibilities, continual harassment and copulation leads to increased water loss rates of female bed bugs (Figure 1), which suggests that individuals are more susceptible to dehydration with increased mating. This increased water loss could cause dehydration stress between bloodmeals, resulting in increased oxidative stress and other types of dehydration-induced damage which will need to be repaired at the expense somatic maintenance [137]. Previous studies failed to reveal an apparent advantage to multiple copulation for females through traumatic insemination [32,152]. It was suggested that the presence of the mesospermalage may select sperm that is better suited for producing offspring [33], but this is still speculative. Recently, it has shown that ejaculate components delay reproductive senescence [38], but this is independent of the number of copulations.

Along with adaptations that occur in females to ameliorate damage from traumatic insemination, avoidance by behavioral or biochemical cues has been documented in fifth instar nymphs, males and females adults. First, females can behaviorally prevent males from accessing the ectospermalge by pressing right side of the abdomen (ectospermalage is located on the ventral side of the abdomen) against a substrate [33]. Second, recently fed females are no longer drawn to aggregation pheromones associated with bed bug harborages, possibly reducing female exposure to harassment from males in commonly utilized off-host harborages [44]. Lastly, there are chemicals cues that prevent unwanted copulation [67,68,70]. Previous studies have shown that males remain on fed females for over 90 seconds and on fed males for less than 10 seconds [33]. This rapid recognition is likely due to chemical factors released from male bed bugs, and recently it was shown that males release alarm pheromones, (E)-2-hexenal and (E)-2-octenal, to reduce mounting [67]. Nymphs release these same two aldehydes to prevent unwanted male copulation [68]. Another recently identified chemical, 4-oxo-(E)-2-hexenal, released from the dorsal abdominal glands of nymphs reduces mating [68,70]. Thus, bed bugs have developed behavioral and pheromonal cues to prevent, or reduce, unwanted mounting.

### 4.4. Microbial and Predator Stress

Previous reviews have addressed the role of predators and disease on the survival of bed bugs [2,153]. Many insects and other terrestrial arthropods could act as predators of bed bugs, such as spiders, mites, ants, pseudoscorpions, could act as predators [2], but their ecological relevance is not known. Exposure to specific pathogens, such as the fungus, *Aspergillus flavus*, and bacteria, *Serratia* sp., have been shown to cause considerable mortality in laboratory populations [2], but whether similar situations occur in field populations is not known. Additionally, as mentioned before, traumatic insemination increases the potential of microbial infection due to cuticle penetration [34,154].

### 4.5. Mechanical Host Response

The mechanical host response (direct contact by the host) could potentially lead to immediate and significant, likely mortality-causing, damage to the bed bugs. To avoid this fate, bed bugs usually feed during the scotophase [2,58], except when severe starvation prompts random or chemical-initiated host seeking. Additionally, while feeding, movement of the host prompts bed bugs to terminate feeding, and then resume feeding after host movement ceases [2]. This interruption in feeding by host movement is the typical reason for multiple bites found in close proximity, likely from the same bug feeding multiple times between interruptions. Lastly, mechanical disturbance of bed bug populations leads to the release of alarm pheromones, prompting bed bugs to evacuate their microhabitat [64,65]. Thus, behavior of bed bugs during feeding reduces the likelihood of host detection.

### 4.6. Potential Stress Associated with Bed Bug Harborages

Bed bugs are thigmotactic, meaning tactile stimulus causes a change in movement. Positive stimulus results in attraction and/or arrestment to a surface and negative results may cause the individual to be repelled. Many insects, such as cockroaches, are known to be thigmotactic. Thus, if bed bugs cannot locate a harborage with proper thigmotactic cues, individuals may become stressed. Additionally, as bed bugs utilize a limited number of harborages that meet their thigmotactic criteria aggregation pheromones will begin to accumulate in these locations, leading to larger aggregations [2,44]. Although these aggregations will decreases water loss rates [39], individuals may defecate on other nearby bed bugs, possibly causing stress by blocking spiracles that could leads to suppressed respiration. This may be a reason why bed bug spiracles are located on underneath their abdomen [2]. Thus, thigmotactic responses of bed bugs may be positive due to increased clustering, which reduces water loss rates and allows mate access. Alternatively, there may also be negative consequences to thigmotactic responses of bed bugs such as the inability to locate thigmotactically-favorable harborages, increased traumatic insemination due to increased mate access and feces potentially blocking spiracles may yield stress.

### 4.7. Pesticide Resistance in Bed Bugs

One of the most critical periods of stress for bed bugs is following the application of insecticides, and survival depends on detoxification and development of permanent resistance. Recently, transcriptomic analysis of bed bugs has identified potential genes that could be critical for detoxification following pesticide exposure [28]. Expression of two detoxification genes, cytochrome P450 and glutathione-*S*-transferase, were specifically tested in control (no pesticide exposure) and pesticide-exposed populations, but only cytochrome P450 was found to be higher in pesticide-exposed populations [28]. Future studies will need to be conducted to establish the role of other detoxification and antioxidant enzymes during pesticide exposure in bed bugs. Specific resistance to pyrethroids has been identified as the result of point mutations in the open reading frame of voltage-sensitive sodium channel genes [30], and resistance mutations are likely to be widespread [16]. Varying levels of resistance to other pesticides, such as DTT, has been reported in bed bug populations [155–160]. Bed bugs avoid harborages treated with certain pesticides, representing a behavioral mechanism of pesticide avoidance [71]. More detailed information on pesticide resistance of bed bugs is provided in this issue of *Insects* [161,162].

## 5. Conclusions and Future Directions

Research on bed bugs had been nearly at a standstill since the 1950s [1,2]. The reemergence of bed bugs has prompted a flurry of research on all aspects of bed bug biology that have built on early studies. Since the last review on bed bugs by Reinhardt and Siva-Jothy [1], significant advances have been made on nearly every facet of bed bug biology. One important field of bed bug biology that has expanded is stress tolerance, which can range from environmental stress to pesticide resistance. Based on current studies, I have included a synopsis of potential stresses that bed bugs will be exposed to in their lifetime and studies that have focused on each topic (Table 2). In relation to human contact, these stresses can be divided into those that will commonly occur (blood feeding, dehydration between bloodmeals and traumatic insemination) to those that occur sporadically (pesticide application and microbial infection). Other than multiple studies that focus on traumatic insemination, few studies have addressed underlying biochemical, molecular and physiological mechanisms of bed bug stress tolerance. To do so, transcriptome studies will be needed to establish *C. lectularius* transcript libraries, eventually leading to the organization of the bed bug genome. After genome annotation, large scale transcriptome projects can be quickly conducted and analyzed to begin to determine underlying transcript changes associated with different physiological states in bed bugs. Thus, progression of research on stress tolerance, along with other aspects of bed bug physiology, will be greatly expanded by developing genomic tools and databases available for *C. lectularius* and other cimcids.

## Figures and Tables

**Table 1:** Cold, heat and dehydration tolerance of the common bed bug, *Cimex lectularius*, and the tropical bed bug, *C. hemipterus*. DT, dehydration tolerance; WLR, water loss rate at 0% relative humidity (RH), 25 °C. Survival for bed bugs at 0 and 75% RH were at 25 °C. The short-term upper and lower lethal limits represent significantly reduced survival after 1–2 h. The long-term upper and lower lethal limits are indicative of reduced survival when individuals are continually held at these temperatures. Results for *C. hemipterus* were from Omori [142], Usinger [2] and How and Lee [42,43] and *C. lectularius* were from Johnson [41], Mellanby [143], Usinger [2], Benoit *et al.* [29,39,40] and Naylor and Boase [143].

*C. hemipterus*	**Water Balance Characteristics**				**Thermal Tolerance**			

	**DT (%)**	**WLR (%/h)**	**Survival (d) at 0% RH**	**Survival (d) at 75% RH**	**Heat**		**Cold**	

					**Short-Term**	**Long-Term**	**Short-Term**	**Long-Term**
Egg	ND	ND	ND	5.8 ±0.2	ND	37–39 °C	ND	<0 °C
1^st^ instar	35–40%	ND	ND	26.1 ±0.8	42–44 °C	30–35 °C	ND	<0 °C
Male	35–40%	ND	ND	32.0 ±2.9*	40–45 °C	30–35 °C	ND	<0 °C
Female	35–40%	ND	ND	62.4 ±3.8*	40–45 °C	30–35 °C	ND	<0 °C
*C. lectularius*								

Egg	24.6 ±3.4	0.037 ±0.001	5.4 ±2.3	5.1 ±4.5	>40 °C/60 °C^1^	37–39 °C	−17°C	<0 °C
1^st^ instar	37.4 ±4.6	0.402 ±0.011	3.9 ±0.9	11.2 ±2.1	40–46 °C	28–33 °C	ND	<0 °C
Male	32.9 ±0.9	0.101 ±0.007	13.6 ±0.8	37.8 ±5.6*	40–46 °C	28–33 °C	ND	<0 °C
Female	34.9 ±1.5	0.402 ±0.013	16.0 ±1.5	72.3 ±3.4*	44–46 °C	28–33 °C	−14 to −16 °C	<0 °C

Note:

* unmated. ND, not determined.

1 > 40 °C exposure was conducted for 30 min in a dryer and 60 °C was in a laundry wash cycle, which were effective against all bed bug stages [142].

**Table 2:** Common stresses that bed bugs (*Cimex lectularius* or *C. hemipterus*) encounter throughout their lifetime. +: indicates this stress has been studied; −: indicates this stress has not be studied.

**Stress**	***C. lectularius***	**Reference**	***C. hemipterus***	**Reference**
Environmental				
Cold	+	[2, 29, 41,143]	+	[2,142]
Heat	+	[2, 29, 41,143,146]	+	[2,41–43,142,145]
Dehydration	+	[2,39–41,145]	+	[42,142]
Blood feeding	+	[111]	−	NA
Microbial stress	+	[2,153]	−	NA
Traumatic inseminaton	+	[32–37,46,67,68,145,154]	+	[2,33]
Thigmotactic response	+	[2]	−	NA
Pesticide resistance	+	[15,16,30,71,155–159]	+	[12,160]
